# First assessment of the prevalence of haemosporidian infections in Accipitriformes raptors in Greece

**DOI:** 10.1007/s00436-024-08445-1

**Published:** 2025-01-07

**Authors:** Grigorios Markakis, Vaidas Palinauskas, Justė Aželytė, Isaia Symeonidou, Viltė Sutkaitytė, Athanasios I. Gelasakis, Anastasia Komnenou, Elias Papadopoulos

**Affiliations:** 1 School of Veterinary Medicine, Faculty of Health Sciences, Aristotle University of Thessaloniki, 54124 Thessaloniki, Greece; 2 Nature Research Centre, Akademijos2, 09412 Vilnius, Lithuania; 3 School of Animal Biosciences, Agricultural University of Athens, 11855 Athens, Greece

**Keywords:** Haemosporidians, Raptors, Greece, *Leucocytozoon*, *Plasmodium*

## Abstract

Haemosporidians, a group of vector-borne parasites that parasitize the blood cells and internal organs of various animal species, are reported to cause severe pathology in raptors. Species belonging to the genera *Plasmodium*, *Haemoproteus* and *Leucocytozoon* are the ones of greatest wildlife importance. The common buzzard (*Buteo buteo*) and the Eurasian sparrowhawk (*Accipiter nisus*) are the most numerous raptor species in Europe. Reliable data is lacking for many raptor species in Greece. The aim of this study was to assess, for the first time, the prevalence and geographical distribution of haemosporidian infection (mainly *Leucocytozoon* and *Plasmodium*) in these two avian species in Greece, in correlation with the risk factors of age and sex. In total, 62 common buzzards and 26 Eurasian sparrowhawks were included in this study, all being admitted for treatment at a Greek Wildlife Rehabilitation Center. Blood samples were collected and microscopical analysis was performed after staining blood smears with Giemsa. DNA was extracted from each sample and a fraction of the mitochondrial cytochrome b gene was amplified by a nested PCR protocol. All positive samples were subjected to sequencing. Total prevalence of haemosporidian infection by morphological and molecular examination was 59% and 73.9%, respectively. Binary logistic regression was carried out. The most prevalent infection was by *Leucocytozoon* spp. Most of the samples had mixed infections. The isolated genetic lineages of *Leucocytozoon* spp. were BUBT2, BUBT3, MILVUS01, ACNI1, BUBO01 and MILANS04. The detected genetic lineages of *Plasmodium* spp. were TURDUS1, BT7 and DONANA02. A new genetic lineage, BUTBUT17, was also identified.

## Introduction

The terms “birds of prey” or “raptors” include birds belonging to the orders Accipitriformes, Falconiformes and Strigiformes. The common buzzard (*Buteo buteo*), along with the Eurasian sparrowhawk (*Accipiter nisus*), both belonging to the order Accipitriformes (the biggest group of raptors which includes many endangered avian species in the world), is the most numerous raptor species in Europe (Svensson 2015; Harl et al. 2022). These birds host many ecto- and endoparasites. Important endoparasites of raptors are haemosporidians (Apicomplexa: Haemosporida), which consist of single-celled eukaryotic protozoa that infect the blood cells and other tissues of organs of birds globally, using blood-sucking dipteran insects as vectors (Hellgren et al. 2004; Chakarov and Blanco 2021; Harl et al. 2022). Species belonging to the genera *Plasmodium*, *Haemoproteus* and *Leucocytozoon* are the ones of greatest wildlife importance, as well as the most widely studied (Valkiūnas 2005; Ings and Denk 2022).

Haemosporidians belonging to the genus *Leucocytozoon* parasitize exclusively avian species, which act as intermediate hosts. Birds get infected after being bitten by black flies, which serve as vectors for the *Leucocytozoon* species, with the exception of the *Leucocytozoon caulleryi*, which is transmitted by midges of the Ceratopogonidae family. The infective stage inside the vectors are sporozoites which enter the vertebrate hosts during the haematophagia (Valkiūnas and Iezhova 2023). Haemosporidians of the genus *Plasmodium* are the causative agents of avian malaria, which is endemic in many areas of the world (Ings and Denk 2022). The hematophagous female mosquitoes of the Culicidae family (mainly species belonging to the genera *Culex*, *Aedes*, *Culiseta* and *Anopheles*) are the vectors of avian malaria parasites. The life cycle of *Haemoproteus* spp. is similar to that of other haemosporidian species. Sporozoites are injected into the bird’s blood circulation by Ceratopogonidae biting midges and Hippoboscidae louse flies, which are final hosts (vectors) for the *Haemoproteus* species (Valkiūnas 2005; Gutiérrez-López et al. 2015; Chakarov and Blanco 2021).

The role of haemosporidiosis in avian pathology, for the time being, cannot be fully estimated. Infection with these causative agents is usually associated with asymptomatic infections as the majority of these protozoa appear quite unharmful and show low pathogenicity (Chakarov et al. 2015; Wiegmann et al. 2021). However, there are recorded cases of birds suffering from clinical symptoms such as anaemia, anorexia, lameness, diarrhoea, dyspnoea and even neurological symptoms, such as convulsions and paralysis (Merino et al. 2000; Knowles et al. 2010; Chakarov and Blanco 2021; Valkiūnas and Iezhova 2023). Recent studies have also revealed that the tissue stages of these parasites can cause impairment in the vital organs of birds (Harl et al. 2022). Moreover, research has shown that haemoprotozoa-infected birds of prey presented to wildlife rehabilitation centres required a longer hospitalization period compared to those being haemoprotozoa-free (Deem 1999; Mohan and Mohan 2017). In addition, some infected birds demonstrate an increased likelihood of having a low body condition score (Chakarov and Blanco 2021). There are also some sporadic reports of birds dying of haemosporidiosis globally. More specifically, one study recorded higher mortality rates in infected raptorial birds than non-infected (Deem 1999). Another survey has recorded deaths in raptors’ chicks infected with *Leucocytozoon* parasites and in owls infected with species of the genus *Haemoproteus* (Remple 2004). Additionally, it is reported that avian blood parasites may potentially cause not only significant declines in bird populations, but also extinctions (Atkinson and Samuel 2010; Dadam et al. 2019). In fact, even if they do not cause direct illness or death, haemosporidians increase the birds’ susceptibility to secondary infections or affect their body condition score and subsequently reduce their ability to reproduce or migrate (Valkiūnas and Iezhova 2023).

The two most effective methods of diagnosing haemosporidiosis are the microscopic examination of stained blood smears and the polymerase chain reaction (PCR)-based technique of blood samples (Valkiunas et al. 2008). PCR methods are supportive to the microscopic ones, as well as they increase the sensitivity of detection and aid the identification of specific lineages (Hellgren et al. 2004; Chakarov and Blanco 2021).

Studying the prevalence and diversity of haemosporidian parasites in wild birds, such as raptors, is of great importance for various reasons. Firstly, the identification of haemosporidians in their erythrocytic phase is helpful for the efficient implementation of many conservation and rehabilitation programs, as they can have a negative effect on the health of many endangered avian species and subsequently on the biodiversity (Valkiūnas 2005; Harl et al. 2022). Another reason is the potential negative effect of haemosporidiosis on aviculture and its important financial consequences (Vakiūnas 2005). Moreover, fowl can get infected with parasites of sympatric wild birds and may cause severe haemosporidiosis; such cases have been recorded to occur in poultry in some countries (Valkiūnas and Iezhova 2023). Finally, the migratory avian species may serve as reservoirs of parasites through the continents (Hellgren et al. 2007; de Angeli Dutra et al. 2021). However, data is currently limited worldwide and lacking in Greece, which is a country posing a crossroad to the migration of birds (Briedis et al. 2020).

The objectives of this study were to determine, for the first time, the prevalence and lineage diversity of haemosporidian infections, mainly focusing on *Leucocytozoon* and *Plasmodium* infections, of common buzzards and Eurasian sparrowhawks in Greece and to subsequently correlate them with their age and sex. The birds’ biology, region of origin and their migration abilities are also discussed in the present study.

## Materials and methods

This study involved 88 accipitriform birds, more precisely 62 common buzzards and 26 Eurasian sparrowhawks. These birds were admitted for treatment to the Wildlife Rehabilitation Center of “ANIMA, Association for Wildlife Care and Protection” in Athens, Greece. All the patients admitted for rehabilitation are recorded in an electronic database along with all the accompanying information and history, including the animal’s age, sex and region of origin.

Immediately after their admission, blood samples (100 µL) were collected from each bird from the brachial or the jugular vein. They were subsequently stored in the SET buffer (0.015 M NaCl, 0.05 M Tris, 0.001 M ethylenediaminetetraacetic acid, pH 8) as a preservative (1:10) and then retained at − 20 °C. Moreover, blood smears were prepared, air dried, fixed with methanol and then stained with Giemsa for the morphological identification of the parasites as well as for the assessment of the intensity of the infection. In detail, a skilled parasitologist examined 100 fields at low magnification (× 400), and then at least 100 fields at high magnification (× 1000) using a Motic BA210 Phase Contrast microscope. Overall, approximately 5 × 10^5^ red blood cells were screened in each blood film (Valkiunas et al. 2008). Intensity of infection was calculated by counting the parasites per 10,000 erythrocytes, as Godfrey et al. suggested (1987). Data on birds’ age, sex and area of origin was collected together with the samples.

DNA was extracted from the blood samples following the manufacturer’s instructions of the Quick-DNA™ Miniprep Kit by Zymo Research. Samples were then screened for positive infections following a modification of the nested PCR protocol (Hellgren et al. 2004), developed by Perez-Rodríguez et al. (2013), which amplifies a fragment of the mitochondrial cytochrome b gene of parasites belonging to *Leucocytozoon* and *Plasmodium* and some *Haemoproteus* parasites. Specifically, these primers are not suitable to detect some specific Haemoproteidae to Accipitriformes raptors (Harl et al. 2024). For this reason, the main focus of this study is on *Leucocytozoon* and *Plasmodium* parasites. The protocol involves a first PCR step using the primers Plas1F (5′-GAGAATTATGGAGTGGATGGTG-3′; Duval et al. 2007) and HaemNR3 (5′-ATAGAAAGATAAGAAATACCATTC-3′; Hellgren et al. 2004), followed by a nested PCR step with the internal primers 3760F (5′-GAGTGGATGGTGTTTTAGAT-3′; Beadell et al. 2004) and the HaemJR4 (5′-GAAATACCATTCTGGAACAATATG-3′).

Those that were positive were subjected to a second nested PCR using initially the primers HaemNFI (5′-CATATATTAAGAGAAITATGGAG-3′) and HaemNR3 (5′-ATAGAAAGATAAGAAATACCATTC-3′) to amplify parasite mtDNA and then HaemF (5′-ATGGTGCTTTCGATATATGCATG-3′) and HaemR2 (5′-GCATTATCTGGATGTGATAATGGT-3′) primers (Bensch et al. 2000) for *Plasmodium* spp. and *Haemoproteus* spp. (Hellgren et al. 2004). PCR products were sequenced afterwards using Big Dye Terminator V3.1 Cycle Sequencing Kit and ABI PRISMTM 3100 capillary sequencing robot (Applied Biosystems, Foster City, CA, USA). We used Geneious (version 2023.1.2) to align and trim the obtained sequences. The mitochondrial cytochrome b gene fragments (478 bp) of haemosporidian parasites were compared with the genetic lineages of parasites available in the NCBI Genbank and the MalAvi database (Bensch et al. 2009). Sequences without any match in either the NCBI Genbank or the MalAvi database were considered new genetic lineages of haemosporidian parasites. Haemosporidian co-infections were determined by visual “double bases” records in the sequence electropherogram.

To map the phylogenetic position of the sequenced lineages, we constructed Bayesian analysis with lineages obtained in our study (*N* = 11). For better determination of sequence position, some lineages of *Leucocytozoon* and *Plasmodium*, which are linked to morphological species (*N* = 5) and parasite lineages found in other studies on raptors mentioned in the discussion (*N* = 5), were added to the analysis. In total, for the construction of the phylogenetic tree, we used 16 *Leucocytozoon* and 5 avian *Plasmodium* lineages. A human malarial parasite, *Plasmodium falciparum*, was used as an outgroup. The selection of best-fit models of nucleotide substitution for phylogenetic analysis was conducted using jModelTest version 2.1.10 (Darriba et al. 2012), and GTR + I + G was selected as the best evolutionary model. The analysis was run for 5 mln generations, with a sampling frequency of every 100th generation. Before constructing a majority consensus tree, 25% of the initial trees in each run were discarded.

### Statistical analysis

All the data from the molecular and microscopical analyses (Table 1) were incorporated in the statistical analysis. Binary logistic regression was used to evaluate species (2 levels, 0 = *Buteo buteo*, 1 = *Accipiter nisus*), age (2 levels, 0 = young, 1 = adult) and sex (2 levels, 0 = male, 1 = female) as potential risk factors and the likelihood that a bird is positive to (i) at least one of the studied haemosporidian species, (ii) specific haemosporidian genera (i.e. *Plasmodium* spp., *Leucocytozoon* spp.) and (iii) mixed haemosporidian infections (Table 2).

**Table 1:**
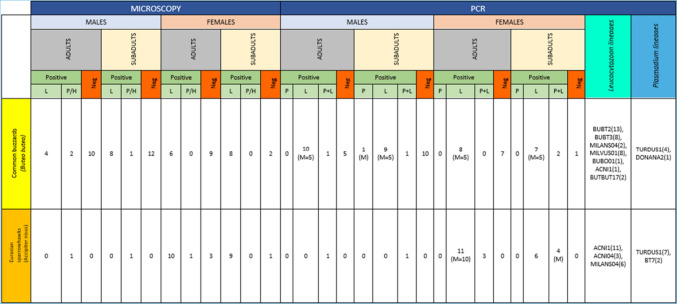
Summary of the molecular and microscopical results (L: infection by *Leucocytozoon* spp., P: infection by *Plasmodium* spp., P + L: mixed infection by *Leucocytozoon* spp. and *Plasmodium* spp., M: mixed infection by different lineages of the same haemosporidian genera, P/H: infection by *Plasmodium* spp. or *Haemoproteus* spp., Neg: negative)

**Table 2:** Associations between the studied risk factors forced into the binary logistic regression model (species, age, and sex) and the likelihood of PCR-positivity to (i) at least one haemosporidian species, (ii) the studied haemosporidian genera (i.e. *Leucocytozoon* spp., and *Plasmodium* spp.) and (iii) mixed infections by these genera

		B^a^	S.E.^b^	Wald	*p*	Odds ratio	95% C.I.^c^ for Odds ratio	
							Lower	Upper
***Infected by at least one haemosporidian species***								
Species	*Buteo buteo*	− 20.5	7868.20	0.00	0.998	0.00	0.00	-
	*Accipiter nisus*	*Ref.*^d^						
Age	Young	0.21	0.54	0.15	0.703	1.23	0.43	3.51
	Adult	*Ref*						
Sex	Male	− 0.41	0.55	0.54	0.463	0.67	0.23	1.97
	Female	*Ref*						
	Constant	21.16	7868.20	0.00	0.998			
***Infected by Leucocytozoon* spp*.***								
Species	*Buteo buteo*	− 20.5	7868.39	0.00	0.998	0.00	0.00	-
	*Accipiter nisus*	*Ref .*^4^						
Age	Young	0.08	0.53	0.02	0.881	1.08	0.38	3.07
	Adult	*Ref*						
Sex	Male	− 0.50	0.55	0.81	0.368	0.61	0.21	1.79
	Female	*Ref*						
	Constant	21.21	7868.39	0.00	0.998			
***Infected by Plasmodium* spp*.***								
Species	*Buteo buteo*	− **2.46**	**0.900**	**7.44**	**0.006**	**0.09**	**0.02**	**0.50**
	*Accipiter nisus*	*Ref.*^d^						
Age	Young	1.03	0.666	2.39	0.122	2.80	0.76	10.33
	Adult	*Ref*						
Sex	Male	0.82	0.910	0.81	0.367	2.27	0.38	13.53
	Female	*Ref*						
	Constant	− 1.18	0.543	4.70	0.030	0.31		
***Mixed haemosporidian infection***								
Species	*Buteo buteo*	− **2.21**	**0.711**	**9.70**	**0.002**	**0.11**	**0.03**	**0.44**
	*Accipiter nisus*	*Ref.*^d^						
Age	Young	0.25	0.489	0.26	0.612	1.28	0.49	3.34
	Adult	*Ref*						
Sex	Male	− 0.48	0.518	0.87	0.350	0.62	0.22	1.70
	Female	*Ref*						
	Constant	1.98	0.642	9.52	0.002	7.25		

^a^Beta coefficient

^b^Standard error

^c^Confidence interval

^d^Reference groups

The statistical significance of individual predictors was tested using the Wald χ^2^ statistic of their regression coefficients (βs). Goodness-of-fit was assessed using the Hosmer–Lemeshow (H–L) test, as well as Cox and Snell R^2^ and Nagelkerke R^2^ indices. Statistical significance was set at the 0.05 level (Table 2).

Cohen’s kappa (κ) value and its 95% confidence interval were estimated to determine the agreement between PCR-testing and microscopy results for the diagnosis of haemosporidian infections. The sensitivity and specificity of microscopy were also estimated, considering PCR as the reference testing method.

## Results

The total prevalence of haemosporidian infections, determined by microscopic examination of the blood smears, in both bird species examined, was 59.0% (52/88). The prevalence of infection was 46.7% (29/62) in common buzzards and 88.5% (23/26) in Eurasian sparrowhawks. In all cases, the intensity of the microscopically detected infections was < 1 parasite/20,000 erythrocytes. *Plasmodium* spp. and *Haemoproteus* spp. were not distinguishable microscopically based on morphological criteria due to the extremely low intensity of parasitemia.

As far as the common buzzards are concerned, the prevalence evaluated by microscopy was 41.9% (26/62) for *Leucocytozoon* spp. and 4.8% (3/62) for *Plasmodium* or *Haemoproteus* spp. In detail, out of 37 male and 25 female birds, 15 (40.5%) and 14 (56.0%), respectively, were found positive by microscopy. Among the 31 adult, 4 juvenile and 27 subadult (young) birds examined, 12 (38.7%), 2 (50.0%) and 15 (55.6%), respectively, were found positive.

As regards Eurasian sparrowhawks, the prevalence calculated by microscopy was 76.9% (20/26) for *Leucocytozoon* spp. and 11.5% (3/26) for *Plasmodium* or *Haemoproteus* spp. In detail, out of 2 male and 24 female birds that were examined, 2 (100.0%) and 21 (87.5%), respectively, were tested positive, while out of 15 adult and 11 subadult birds that were examined, 12 (80.0%) and 11 (100%), respectively, were found positive.

**Fig. 1:**
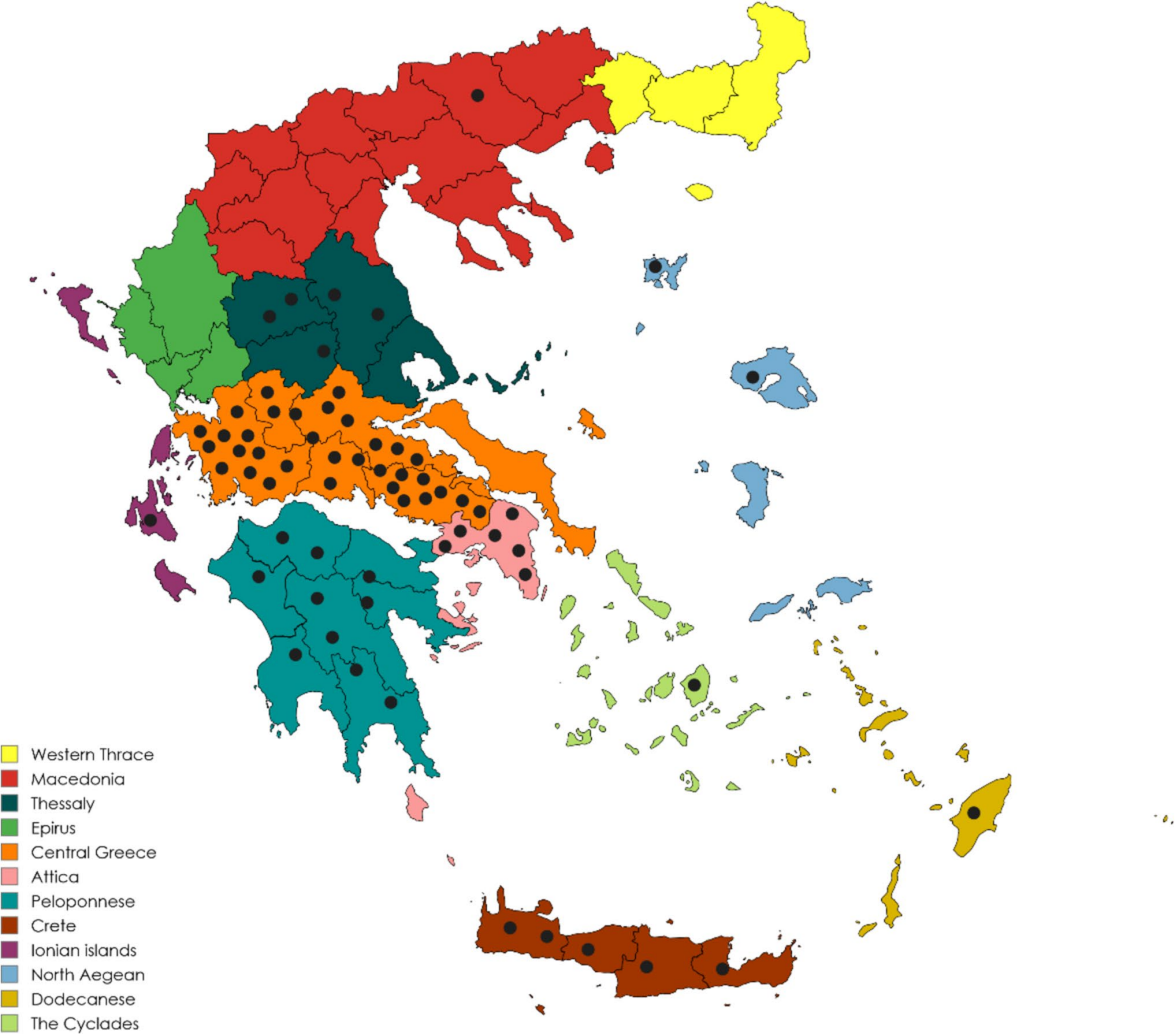
Geographical regions of Greece where samples positive for haemosporidians originated from

In the case of molecular detection of haemosporidian parasites (Table 1), the estimated total prevalence in both of the examined raptorial species was 73.9% (65/88). Specifically, the prevalence of haemosporidian infections was 62.9% (39/62) in common buzzards and 100.0% (26/26) in Eurasian sparrowhawks. The total prevalence for each parasitic genus as detected by PCR was 72.7% (64/88) for *Leucocytozoon* spp., with 51.5% (33/64) of the infections being co-infections by different *Leucocytozoon* genetic lineages and 17% for *Plasmodium* spp. We did not detect any birds infected with *Haemoproteus* spp. using the primers described in the methods section. However, it is important to note that we cannot conclusively state that the birds were free of *Haemoproteus* parasites. A recent study by Harl et al. (2024) has shown that the general primers used in our study may fail to detect certain *Haemoproteus* lineages that are specific to Accipitriformes raptors. Therefore, our results focus solely on *Leucocytozoon* and *Plasmodium* distribution (Fig. 1).

**Fig. 2:**
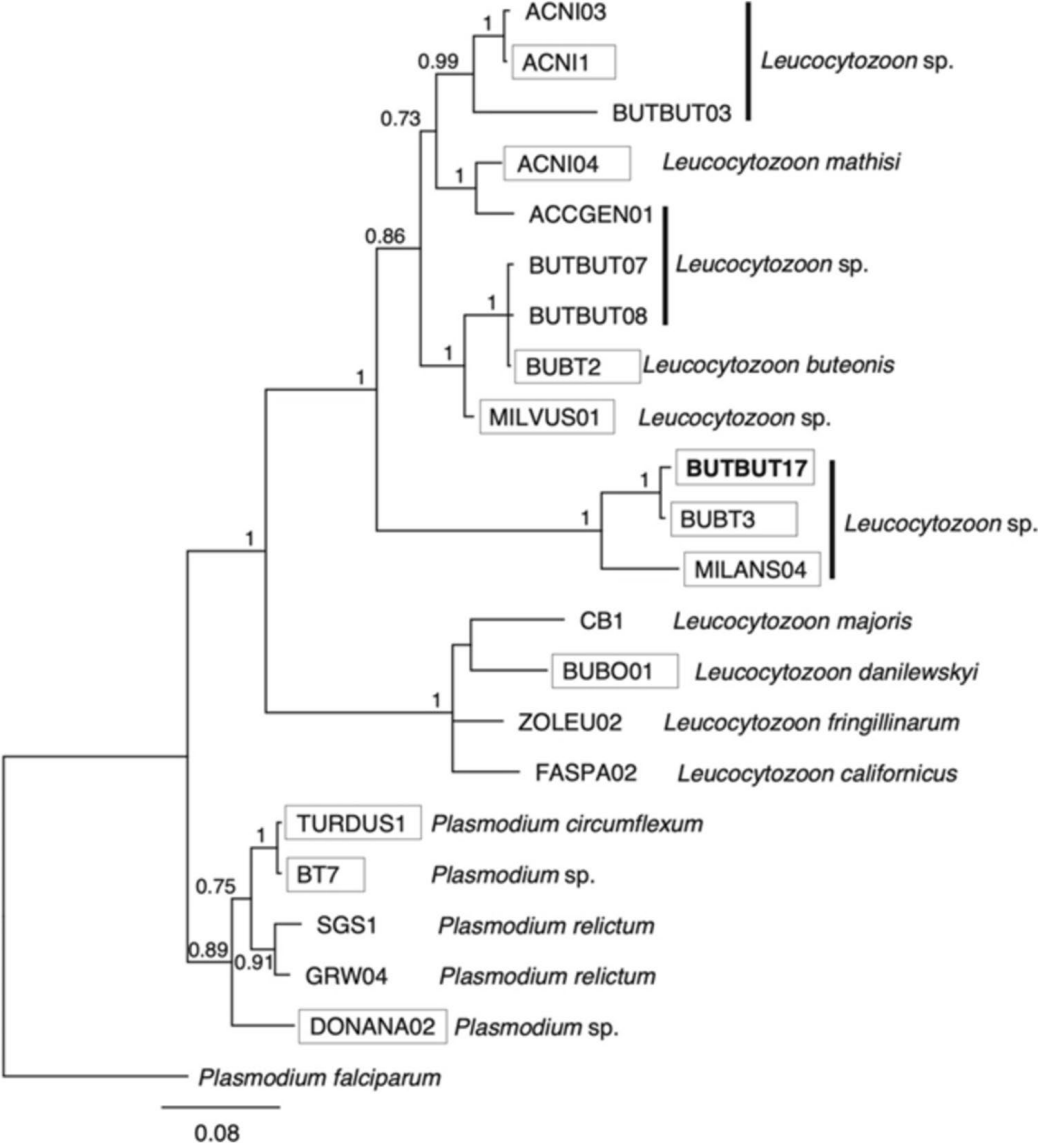
Bayesian phylogenetic tree based on mitochondrial cytochrome b gene fragments (478 bp) of 16 *Leucocytozoon* spp. and 5 avian *Plasmodium* spp. A sequence of *Plasmodium falciparum* was used as an outgroup. Lineages of parasites with codes of the MalAvi database are given for each sequence. A grey rectangle indicates the lineages obtained in this study. The bold font marks a new lineage

In common buzzards, the prevalence detected by PCR was 59.7% (39/62) for *Leucocytozoon* spp. and 9.7% (6/62) for *Plasmodium* spp. The 64.1% (25/39) of the infections were mixed; οf these infections, 20.0% (5/25) were infections by *Leucocytozoon* spp. and *Plasmodium* spp., and 80.0% (20/25) were infections by various *Leucocytozoon* lineages. Out of 37 male and 25 female birds examined, 22 (59.5%) and 17 (68.0%), respectively, were PCR-positive for haemosporidians. Among the 31 adult, 4 juvenile and 27 subadult birds examined, 19 (61.3%), 1 (25.0%) and 17 (62.9%), respectively, were found PCR-positive for haemosporidians. The detected genetic lineages were BUBT2 (*Leucocytozoon buteonis*), BUBT3, MILVUS01, MILANS04, BUBBO01 and ACNI1 of *Leucocytozoon* spp. Other detected genetic lineages belonged to the *Plasmodium* genus, e.g*.* TURDUS1 (*Plasmodium circumflexum*) and DONANA02. A new genetic lineage, BUTBUT17, of *Leucocytozoon* sp. was also detected in two individuals (lineage code BUTBUT17 in MalAvi database, GenBank number PQ474641; Fig. 2) and the gametocytes were also detected microscopically.

As for the Eurasian sparrowhawks, they were all infected by *Leucocytozoon* spp. (100.0%; 26/26), while the prevalence for *Plasmodium* spp. was 34.6% (9/26). The 80.8% (21/26) of the infections were mixed, with 42.9% (9/21) of them being coinfections by *Leucocytozoon* spp. and *Plasmodium* spp. and the rest 57.2% (12/21) being mixed infections by various *Leucocytozoon* lineages. The detected genetic lineages were ACNI04 (*Leucocytozoon mathisi*), ACNI1 and MILANS04 of *Leucocytozoon* spp. and TURDUS1 and BT7 of *Plasmodium circumflexum* (Fig. 2). 

Eurasian sparrowhawks were 11.1 times more likely to be infected by *Plasmodium* spp. compared to common buzzards (95% CI, 2.0–50.0; *p* < 0.01). Moreover, the likelihood of Eurasian sparrowhawks being infected by a mixed haemosporidian infection was increased by 9.1 times compared to common buzzards (95% CI, 2.3–33.3; *p* < 0.01). In all cases, age and sex had no significant effect on haemosporidian infection. The effect of bird species was not possible to be estimated when considering the infection by at least one haemosporidian parasite as well as the infection by *Leucocytozoon* spp. because none of the studied *Accipiter nisus* was found to be PCR negative.

Overall, a moderate but statistically significant agreement between PCR testing and microscopy for the diagnosis of haemosporidian infections was observed (κ = 0.656). The estimated sensitivity [True positives / (True positives + False negatives)] and specificity [True negatives / (True negatives + False positives)] of microscopy was 78.5% and 100.0%, respectively, when PCR was considered the reference testing method.

## Discussion

For the purpose of our study, the two most common Accipitriformes species, the common buzzard (*Buteo buteo*) and the Eurasian sparrowhawk (*Accipiter nisus*), were tested for haemosporidian parasites by microscopy and using molecular methods for the first time in Greece.

Ten *Leucocytozoon* species, six *Haemoproteus* species and only three species of *Plasmodium* have been described in accipitriform birds including the recently described *H. multivacuolatus* in common buzzards (Harl et al. 2022; 2024). The prevalence of haemosporidian parasites varies significantly between different species and habitats, and even between populations of the same species (Valkiūnas and Iezhova 2023). This variation is influenced by three main factors: the vectors, the parasites themselves and the vertebrate hosts (Svobodová et al. 2015). The effect of the host can be seen by the detected significant difference between the prevalence of infection in the nocturnal raptorial species, 30.5%, and the diurnal ones, 46.3% (Muñoz et al. 1999). Furthermore, Lierz et al. (2002) have presented a general overview on parasites of Germany’s birds of prey, whereas the common buzzards presented the highest prevalence (44.8%) in blood parasites (*Leucocytozoon* and *Haemoproteus*).

It is also important to mention the significance of the examination method used in the rational assessment of the results. In the present study, although an agreement between PCR and microscopy was detected, still microscopy presented lower sensitivity than PCR. It is established that the molecular methods offer higher sensitivity in detecting haemosporidian infections, as microscopy of blood smears may underestimate the prevalence of haemosporidians, particularly in cases of low parasitaemia (Hellgren et al. 2004; Ortego et al. 2007; Pérez-Rodríguez et al. 2013) or lower quality of the blood smears. By complementing microscopical analysis with molecular techniques to detect low intensities of *Plasmodium* spp. and *Haemoproteus* spp. alongside *Leucocytozoon* spp., the sensitivity of pathogen detection increases. However, molecular methods have some limitations too. It is well known in avian haemosporidian research that cases of mixed infections are often underestimated, as selective amplification of DNA may occur, with some parasites causing higher parasitaemias, which can obscure the DNA of other parasites (Valkiūnas et al. 2006). The application of more than one PCR protocol can be helpful for the detection of parasite lineages in mixed infections (Hellgren et al. 2004; Bernotiene et al. 2016). Additionally, it has been recently published that primers previously considered general for *Haemoproteus* parasites do not accurately reflect the true prevalence of *Haemoproteus* in raptor species, namely in *Accipiter* birds, as demonstrated in a recent study by Harl et al. (2024). It is noteworthy that in a study conducted by Krone et al. (2008) regarding the haemosporidians of the European birds of prey, almost the same prevalence of infection was detected both by microscopy and using molecular methods (19.8% and 21.8% respectively). Harl et al. (2022) also screened Accipitriformes raptors by nested PCR, and the total prevalence of infection detected was 44.0%; however, microscopic examination was not used in that survey.

In the present study, coinfections between species of the studied genera (*Leucocytozoon* and *Plasmodium*) were detected. More specifically, coinfections of *Leucocytozoon* spp. and *Plasmodium* spp. were present in 16.6% of common buzzards and 42.9% of Eurasian sparrowhawks. However, in both bird species, mixed infections by different *Leucocytozoon* lineages were more prevalent. This is in accordance with the results of a study conducted by Svobodová et al. (2015) in the Czech Republic, regarding the same raptorial species. In that study, mixed infections with *Leucocytozoon* and *Trypanosoma* parasites outnumbered those by *Leucocytozoon* and *Haemoproteus*, and this is explained by the fact that the latter have different vectors, despite being phylogenetically closely related parasites. Similarly, in a 7-year prospective study conducted by Svobodová et al. (2023) in the Czech Republic, Eurasian sparrowhawks were screened for haemosporidian parasites, with most of the infections detected being mixed. The most prevalent single infection was the one caused by *Leucocytozoon* spp.

Regarding the distribution of haemosporidian infections, our study detected infected birds in 9 of the 13 geographical regions of Greece: Central Macedonia, Thessaly, Ionian Islands, Continental Greece, Attica, Peloponnese, North Aegean, South Aegean and Crete. None of the birds that were involved in the current study originated from Eastern Macedonia and Thrace, Epirus, Western Greece or Western Macedonia (Fig. 1). However, given the wide geographical distribution of cases and the proximity of the studied regions to these areas, it is reasonable to speculate that infected birds may also be present in the non-studied territories.

### *Leucocytozoon* spp.: prevalence, species and associated risk factors

In the current study, raptors were found to be most frequently infected by *Leucocytozoon* spp., with the prevalence of infection being remarkably high (72.7%). *Leucocytozoon* species are considered cosmopolitan parasites, as they are thriving in countries with not only hot climates, but in cold as well, posing a global threat for many bird species, including poultry (Valkiūnas and Iezhova 2023). The vectors spreading *Leucocytozoon* can cover large distances (Chakarov and Blanco 2021). This probably explains the high prevalence of these parasites (approximately 100% in some raptor species) in both wild and domestic birds. In the same context, the high prevalence of *Leucocytozoon* infection that was detected in this study can be explained by a high distribution of the black flies in the studied regions of Greece (Otranto et al. 2012).

In accordance with the present study, Muñoz et al. (1999) examined blood samples from raptorial birds in Catalonia using microscopic examination and identified *Leucocytozoon* as the most frequently detected genus. Likewise, protozoa belonging to the *Leucocytozoon toddi* group were the most common haemosporidian parasites detected using molecular methods in Accipitriformes in a survey conducted in Austria (Harl et al. 2022). In the same frame, *Leucocytozoon* spp. presented the highest occurrence among haemosporidian parasites in a 7-year study of Eurasian sparrowhawks in Prague, Czech Republic (Svobodová et al. 2023).

Regarding the prevalence of *Leucocytozoon* spp. in Eurasian sparrowhawks, two studies have demonstrated extremely high infection rates, i.e. 91.6% in Spain (Muñoz et al. 1999) and 93.0% in the UK (Peirce et al. 1983). Regarding common buzzards, a long-term study conducted in Germany revealed a 40.0% prevalence of infection by *Leucocytozoon toddi* (Chakarov et al. 2008).

Infections with a single species of *Leucocytozoon* are not common. As a result, molecular characterization is often challenging due to the high frequency of mixed infections (Valkiūnas and Iezhova 2023). This is in accordance with the results of the present study, as approximately one out of two infected birds was co-infected by different *Leucocytozoon* genetic lineages.

All ten *Leucocytozoon* lineages that have been isolated from accipitriform raptors belong to the *L. toddi* group (Harl et al. 2022). The most prevalent *Leucocytozoon* species identified in the present study were *Leucocytozoon buteonis*, *Leucocytozoon mathisi*, and *Leucocytozoon toddi*. Infections by previously undescribed species of the genus *Leucocytozoon* have also been identified. In the study conducted by Harl et al. in (2022), the genetic lineages isolated from accipitriform raptor species (listed under a descending order of frequency) were BUBT2, BUTBUT03, BUTBUT07, CIAE03, MILVUS01, ACNI04, MILANS04, BUTBUT08, ACCGEN01 and ACNI1 (Harl et al. 2022). In another study on Eurasian sparrowhawks in Prague, the lineages isolated were, in descending order, the haplotypes ACNI1 and ACNI3 of *Leucocytozoon* spp. and the haplotype ACNI4 of *Leucocytozoon mathisi* (Svobodová et al. 2023).

Regarding risk factors, age, sex and species of birds were assessed for being associated with *Leucocytozoon* spp. infection likelihood. No significant effect on infection with *Leucocytozoon* parasites was observed with regard to age. According to surveys on nestlings, no definite correlation between *Leucocytozoon* spp. infection and age was also detected (Pérez-Rodríguez et al. 2013; Chakarov and Blanco 2021; Svobodová et al. 2023). However, there are some studies which concluded that the likelihood of *Leucocytozoon* infection increased with age in nestlings (Chakarov et al. 2008; Svobodová et al. 2015; Hanel et al. 2016).

Sex was not found to be a risk factor in the current study, as no significant effect on molecular positivity in *Leucocytozoon* spp. was detected. This is consistent with several other studies, which also concluded that there was no effect of sex on the prevalence of haemosporidian infections, and therefore, it is not considered to be a risk factor (Peirce et al. 1983; Lei et al. 2013; Pérez-Rodríguez et al. 2013; Chakarov and Blanco 2021; Svobodová et al. 2023). This is interesting, as it seems that a “parent-to-offspring” infection pattern has been noted, both in Eurasian sparrowhawks and in common buzzards as well, which states that transmission of the blood parasites takes place to a greater extent during breeding (Chakarov et al. 2015). Furthermore, the excessive production of kairomones by females was speculated to be appealing to the dipterans that transmit these parasites. However, in accordance with the sex semiochemical hypothesis, sex differences are more likely in species with uniparental incubation (Grieves et al. 2022), whereas raptors are generally biparental (Wreford 2014), although not with equal frequencies between sexes.

### *Plasmodium* spp.: prevalence, species and associated risk factors

The prevalence of infection by *Plasmodium* spp. detected in our study (17%) was higher than the prevalence calculated in a study regarding the birds of prey in France (< 8%) (Giorgiadis et al. 2020), while in the Czech Republic, no *Plasmodium* species were detected in Eurasian sparrowhawks screened for blood parasites (Svobodová et al. 2023).

*Plasmodium circumflexum* was the most frequent haemosporidian parasite of the genus *Plasmodium* infecting the birds of prey, which is in accordance with the findings presented by Harl et al. (2022) in Austria. *Plasmodium circumflexum* is not an Accipitriformes-specific parasite. Transmission of malarial parasites between birds belonging to different orders is not uncommon, especially when a variety of avian species is kept in the same place, like in a wildlife rehabilitation centre. In that way, dipteran vectors of haemosporidians can transmit the parasites from one bird to another during sanguinivory, but the birds hospitalized at a rescue centre may also be exposed to insects that might not come across in their natural habitat (Harl et al. 2022). This underlines the necessity to conduct more studies focusing on the morphological confirmation of the parasites’ gametocytes, which will confirm the patency of the infection. By finding gametocytes in the blood, it can be proved that it is not only an abortive development of the parasite in the host, but those parasites develop transmissive stages which means that these birds can be hosts of those parasites. The genetic lineage TURDUS1 which was the most frequently detected in the current study was the same that was most commonly isolated in Austria’s raptors (Harl et al. 2022). This lineage has also been detected in northern goshawks (*Accipiter gentilis*) in the Czech Republic (Hanel et al. 2016), as well as in the 31.8% of the Eurasian sparrowhawks and in the 1.9% of the common buzzards in France (Harl et al. 2024). In the same study, the 9.1% of the Eurasian sparrowhawks and the 9.4% of the common buzzards were found infected by *Plasmodium circumflexum* BT7 that was also isolated from two Eurasian sparrowhawks in the present study. These two genetic lineages—TURDUS1 and BT7—are two of the most commonly isolated *Plasmodium* lineages in raptors of the order Accipitriformes (Harl et al. 2024).

Age and sex showed no significant effect on infection by *Plasmodium* spp. Although a few studies suggest that age and sex have no significant effect on the prevalence of *Plasmodium* infections (Pérez-Rodríguez et al. 2013; Chakarov and Blanco 2021), there is still limited data on these factors in relation to *Plasmodium* spp. infections in raptors.

## Conclusion

In this study, we explored the prevalence and diversity of haemosporidian infections in common buzzards and Eurasian sparrowhawks in Greece, focusing primarily on *Leucocytozoon* and *Plasmodium* species. Our findings highlight a high prevalence of *Leucocytozoon* infections, which were more common than *Plasmodium* spp., with mixed infections being frequent in both examined raptor species. No *Haemoproteus* spp. infections were detected, and this result should be interpreted cautiously given the recent discovery that general primers may not detect *Accipiter*-specific *Haemoproteus* lineages.

Our results also show that Eurasian sparrowhawks have a significantly higher likelihood to be infected by *Plasmodium* spp. compared to common buzzards. Host age and sex appeared to have no significant effect on infection by these blood parasites. A new genetic lineage of *Leucocytozoon* sp. was detected in two common buzzards with observed presence of gametocytes, confirming this avian species as a patent host. The aforementioned data and the results of the present study underline the necessity of further investigation of the prevalence, the diversity and the consequences of haemosporidiosis in raptors, a group of birds that is underrepresented in studies of haemosporidians, as well as of the distribution of the insect vectors too.

## Data Availability

No datasets were generated or analysed during the current study.
